# Successful Therapeutic Approach to Facial Hyperpigmentation Secondary to Addison’s Disease: A Case Report and Literature Review

**DOI:** 10.7759/cureus.107884

**Published:** 2026-04-28

**Authors:** Laura A Colorado Franco, Melanie Villamizar, Cindy Lorena Caceres, César González Ardila, Angie Julieth Holguín Molina

**Affiliations:** 1 Dermatology, Clinica Privada Dermatologia, Bogotá, COL; 2 Dermatology, Universidad el Bosque, Bogotá, COL

**Keywords:** addison's disease, adrenal insufficiency (ai), clinical case report, facial hyperpigmentation, melasma, melasma and facial melanosis

## Abstract

Addison’s disease, or primary adrenal insufficiency, is a rare endocrine disorder characterized by deficient glucocorticoid and mineralocorticoid production, frequently associated with cutaneous hyperpigmentation due to chronic elevation of adrenocorticotropic hormone. Facial hyperpigmentation may persist despite adequate hormonal replacement, representing a therapeutic challenge with limited evidence to guide dermatologic management.

We report the case of a 41-year-old female patient with an eight-year history of progressive hyperpigmentation affecting the face, hands, and oral mucosa. Physical examination revealed severe facial involvement with brown-violaceous macules and associated telangiectasias. The patient had a prior diagnosis of autoimmune ovarian failure in 2017 and was diagnosed with Addison’s disease in 2020, receiving stable glucocorticoid replacement therapy. Histopathological evaluation demonstrated basal layer hyperpigmentation without interface dermatitis, consistent with endocrine-related pigmentation and overlapping melasma features.

A multimodal therapeutic approach was implemented, including picosecond Nd:YAG laser therapy (approximately seven sessions at six- to eight-week intervals), oral tranexamic acid (250 mg twice daily), and a topical depigmenting regimen containing tranexamic acid, niacinamide, retinoids, and antioxidants. The patient demonstrated a significant reduction in modified Melasma Area and Severity Index score from 19.8 to 7.8 (60.6% reduction), along with a marked improvement in quality of life, as evidenced by a posttreatment Melasma Quality of Life Scale score of 20/70.

This case highlights that persistent hyperpigmentation in Addison’s disease may not fully resolve with endocrine treatment alone and may require adjunctive dermatologic interventions. A personalized, multimodal strategy targeting multiple pathogenic pathways, including melanogenesis, plasmin-mediated signaling, vascular factors, and dermal pigment deposition, can achieve significant improvement, even in patients with darker phototypes and long-standing disease. These findings underscore the importance of a multidisciplinary approach and suggest a potential role for combination therapies, including laser-based modalities, in managing complex pigmentary disorders associated with systemic conditions. Further studies are needed to establish standardized treatment protocols and evaluate long-term outcomes.

## Introduction

Addison’s disease, or primary adrenal insufficiency, is a rare endocrine disorder characterized by inadequate production of glucocorticoids and mineralocorticoids due to adrenal cortex dysfunction. Among its clinical manifestations, cutaneous hyperpigmentation is one of the most distinctive and often early signs, resulting from chronic elevation of adrenocorticotropic hormone (ACTH) and its melanocyte-stimulating properties [1]. This finding commonly involves sun-exposed areas, mucous membranes, palmar creases, and the face, where it may have a considerable aesthetic and psychosocial impact [2].

Facial hyperpigmentation associated with Addison’s disease may persist despite adequate hormonal replacement therapy, posing a therapeutic challenge for clinicians. While systemic management remains essential to control the underlying endocrine disorder, the optimal dermatologic approach to residual or persistent hyperpigmentation has not been well established. In particular, there is a lack of standardized treatment protocols and limited evidence regarding the efficacy and safety of multimodal therapies, such as the combination of systemic agents, topical treatments, and laser-based modalities, especially in patients with darker phototypes and long-standing disease, in whom pigmentation tends to be more refractory [3].

This article describes the feasibility and clinical outcome of a multimodal dermatologic approach to facial hyperpigmentation secondary to Addison's disease in a single patient, and proposes hypotheses for future studies, highlighting the role of dermatologic intervention as a complement to systemic treatment.

## Case presentation

On April 25, 2025, a 41-year-old female patient, a nurse by profession, presented to a private dermatology clinic in Bogotá, Colombia, with an eight-year history of progressive hyperpigmentation. The initial phase of her disease and early management occurred at an external institution, limiting the availability of detailed records of prior treatments. The condition was characterized by asymmetric, irregular brown-violaceous hyperpigmented macules involving the face, hands, and oral mucosa. Despite standard endocrine management, the patient reported progressive worsening of the pigmentation over time.

**Figure 1:**
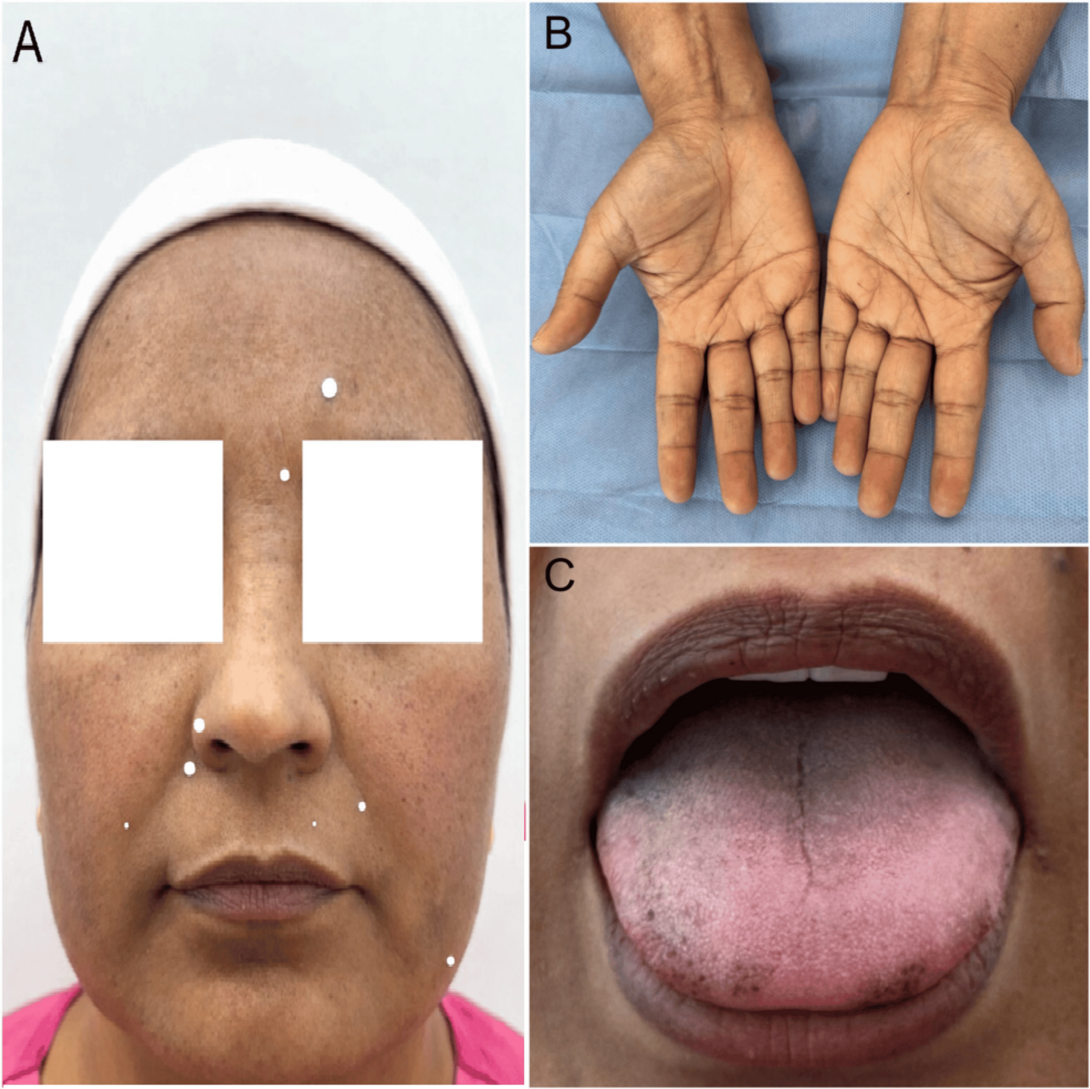
Clinical findings of mucocutaneous hyperpigmentation (A) Diffuse facial hyperpigmentation predominantly involving the malar regions, characterized by confluent dark brown macules with a symmetric distribution. (B) Bilateral palmar hyperpigmentation with marked accentuation of dermatoglyphic creases. (C) Oral mucosal pigmentation showing diffuse involvement of the tongue with focal punctate hyperpigmented macules along the lateral border

Physical examination at the time of evaluation revealed diffuse pigmentation with confluent dark brown-violaceous macules affecting the forehead, cheeks, and chin, corresponding to a severe grade (4/4), where darkness is rated from 0 to 4 according to the modified Melasma Area and Severity Index (mMASI). Additionally, a moderate number of telangiectasias were observed in the bilateral malar regions. The patient had Fitzpatrick skin phototype V, a thickened skin texture, punctate pigmentation along the lateral border of the tongue, and violaceous discoloration of the soft palate. The presence of multiple dental amalgam restorations was also noted (Figure 1).

Disease severity was further assessed using the mMASI, and quality of life was evaluated using the Melasma Quality of Life Scale (MelasQoL), which demonstrated a severe clinical impact at baseline, with an initial mMASI score of 19.8 and a MelasQoL score of 44 of 70.

In 2017, the patient was evaluated by the Endocrinology service and diagnosed with autoimmune ovarian failure. Hormone replacement therapy was initiated with a transdermal estradiol spray solution at 1.53 mg, administered every other day, in combination with medroxyprogesterone acetate 5 mg, half a tablet taken every other day.

In 2020, the diagnosis of primary adrenal insufficiency (Addison’s disease) was established based on low serum cortisol levels, elevated ACTH levels, and confirmatory endocrine testing performed by the endocrinology service. Glucocorticoid replacement therapy was initiated with hydrocortisone 10 mg every 12 hours and deflazacort 3 mg at night, a regimen that has been maintained to the present time.

**Figure 2:**
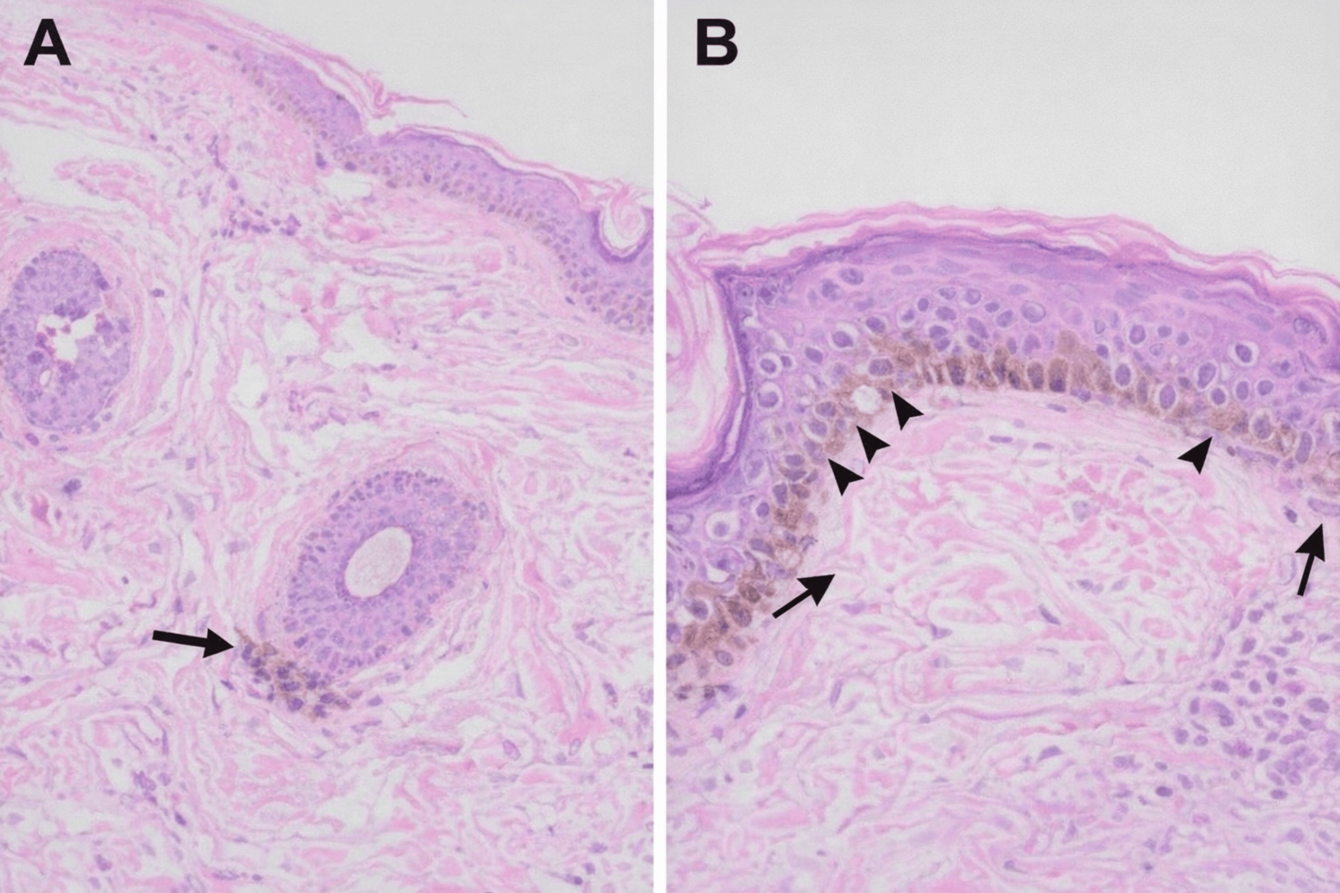
Histopathologic findings (H&E stain) (A) Low-power view demonstrating mild epidermal acanthosis with increased basal layer pigmentation. Focal melanin deposition is also observed surrounding the follicular epithelium (arrow), along with a mild superficial perivascular lymphohistiocytic infiltrate in the dermis. (B) Higher magnification highlighting prominent melanin deposition along the basal layer (arrowheads) without evidence of dermoepidermal interface damage. Scattered superficial dermal inflammatory cells are also present (arrows) H&E: hematoxylin and eosin

To further clarify the diagnosis and guide therapeutic management, a skin biopsy of the left malar region was performed. Histopathological examination revealed epidermis with orthokeratosis, acanthosis, and basal layer hyperpigmentation, without evidence of dermoepidermal interface alteration. The dermis showed a mild superficial perivascular lymphohistiocytic inflammatory infiltrate (Figure 2). Overall, these findings were considered suggestive of pigmentation related to Addison’s disease with superimposed melasma.

Following histopathological confirmation, a multimodal therapeutic approach was initiated. This strategy was selected to target multiple pathogenic mechanisms involved in persistent hyperpigmentation, including increased melanogenesis, dermal pigment deposition, vascular factors, and plasmin-mediated pathways. Treatment included approximately seven sessions of picosecond Nd:YAG laser therapy performed at six- to eight-week intervals (Table 1), aimed at fragmenting dermal and epidermal pigments. In addition, oral tranexamic acid (250 mg twice daily) was prescribed to inhibit plasmin-mediated melanocyte activation and vascular components. A topical depigmenting formulation containing tranexamic acid (3%-5%), niacinamide (4%-5%), a low-concentration retinoid (0.025%-0.05%), and antioxidant agents including ascorbic acid derivatives (1%-10%) and tocopherol (<1%) was also used to further suppress melanogenesis, enhance epidermal turnover, and reduce oxidative stress. The topical therapy was applied twice daily (morning and night) for one month, after which the frequency was reduced to once nightly.

**Table 1:** Multimodal picosecond Nd:YAG laser parameters

Pass	Wavelength (nm)	Spot size (mm)	Fluence (J/cm^2^)	Frequency (Hz)	Shots
Vascular	5-15/LT	10	3	10	1,300
Toning	1,064	7	0.7	10	1,700
Microfractional	1,064	6 × 6	1.25	10	1,300

**Figure 3:**
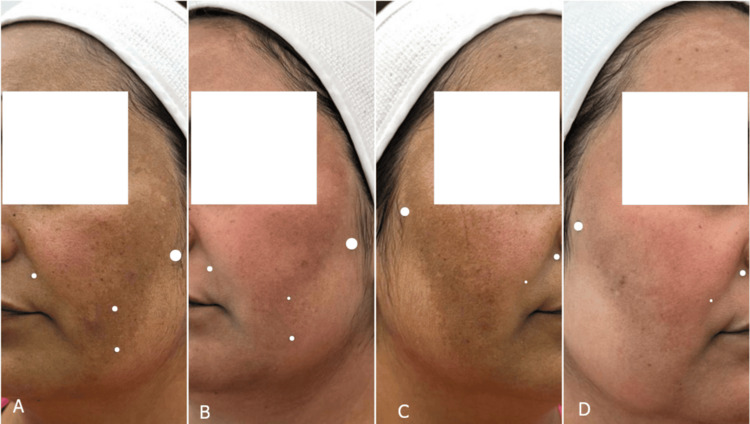
Sequential clinical images demonstrating treatment response (A) Left hemiface before treatment, showing diffuse hyperpigmented macules. (B) Left hemiface after treatment, with noticeable reduction in pigmentation intensity. (C) Right hemiface before treatment, demonstrating confluent brown macules. (D) Right hemiface after treatment, showing significant improvement with decreased pigmentation

During follow-up, clinical improvement was objectively assessed using the mMASI [4,5], in addition to quality-of-life evaluation using the MelasQoL [6]. The patient demonstrated a significant reduction in mMASI score from 19.8 to 7.8 (60.6% reduction) (Figure 3), accompanied by a marked improvement in quality of life, as reflected by a posttreatment MelasQoL score of 20/70.

## Discussion

Cutaneous hyperpigmentation is a hallmark clinical feature of Addison’s disease and results from chronic elevation of ACTH, which shares structural homology with melanocyte-stimulating hormone. This hormonal overlap leads to diffuse melanocytic stimulation and increased melanin production, predominantly affecting sun-exposed areas, mucous membranes, palmar creases, and areas subjected to friction. Facial involvement is particularly common and may represent one of the earliest manifestations of the disease, often preceding systemic symptoms, as described in both endocrine and dermatologic literature [1-3].

According to the Endocrine Society Clinical Practice Guideline, glucocorticoid replacement therapy remains the cornerstone of treatment for primary adrenal insufficiency and usually leads to gradual improvement in hyperpigmentation [1]. However, complete resolution is not universal. Persistent pigmentation may occur despite adequate endocrine control, particularly in patients with long-standing disease, darker skin phototypes, or prolonged exposure to elevated ACTH levels prior to diagnosis. In these cases, residual pigmentation may be related to persistent melanocytic activation and increased melanin deposition within the basal layer, as described in other forms of chronic hyperpigmentation [2,3], although this remains speculative in the context of the present case and may not be fully reversible with hormonal normalization alone. Furthermore, several dermatologic strategies have been proposed for managing persistent pigmentation, including topical depigmenting agents, chemical peels, and energy-based devices such as Q-switched or picosecond lasers. While these approaches may provide partial improvement, clinical outcomes can be variable, and recurrence or postinflammatory hyperpigmentation may occur, particularly in higher phototypes.

The present case illustrates persistent facial hyperpigmentation despite appropriate systemic management of Addison’s disease. Histopathological examination revealed basal layer hyperpigmentation without dermoepidermal interface alteration, along with mild superficial perivascular inflammation. Overall, the findings suggested endocrine-related pigmentation with overlapping melasma-like features, rather than a single isolated pigmentary disorder. This overlap is clinically relevant, as melasma and endocrine-induced hyperpigmentation may coexist, particularly in patients with higher phototypes and chronic ultraviolet exposure, creating diagnostic and therapeutic challenges. Table 2 provides a comparative summary of key features among these conditions.

**Table 2:** Key clinical and histopathologic features of Addison’s disease-related hyperpigmentation and melasma Source: This is an original table created by the authors

Feature	Addison’s disease-related hyperpigmentation	Melasma
Etiology	Elevated ACTH with melanocyte stimulation [1-3]	Multifactorial (UV exposure, hormonal factors, genetics) [4,7,8]
Onset	Insidious, progressive [1,2]	Gradual, often exacerbated by sun exposure [4,7,8]
Distribution	Diffuse, sun-exposed areas, palmar creases, mucosa [1-3]	Symmetric facial involvement (malar, centrofacial) [4-8]
Mucosal involvement	Common [1,3]	Absent [4]
Systemic symptoms	Present (fatigue, weight loss, hypotension) [1,2]	Absent [4]
Fitzpatrick phototype	More evident in darker phototypes [2]	More common in darker phototypes [4-8]
Histopathology	Basal layer hyperpigmentation without interface damage [3]	Epidermal ± dermal melanin deposition [8]
Response to hormone replacement	Partial, often incomplete [1,2]	Not applicable
Response to dermatologic treatment	Adjunctive benefit [1]	Primary treatment modality [4-8]

Currently, there are no standardized dermatologic guidelines for the management of persistent hyperpigmentation secondary to Addison’s disease. Available evidence is limited to expert opinion and isolated case reports, most of which focus on topical depigmenting agents and photoprotection [3]. In contrast, the multimodal therapeutic approach used in this case targeted multiple pathogenic pathways simultaneously, including melanocyte activity, vascular involvement, and dermal pigment deposition. However, the relative contribution of each treatment modality cannot be clearly determined, which represents an inherent limitation of this report. Nevertheless, the sustained clinical response observed supports a possible synergistic effect of the combined therapeutic strategy.

Picosecond Nd:YAG laser therapy was selected due to its ability to target melanin with minimal thermal diffusion, making it particularly suitable for patients with darker skin phototypes and reducing the risk of postinflammatory hyperpigmentation. However, despite its favorable safety profile, the risk of post-inflammatory hyperpigmentation cannot be completely eliminated, particularly in higher phototypes, and should be carefully considered. In this case, no significant adverse effects were observed. Laser-based therapies, especially Nd:YAG platforms, have increasingly been used as adjunctive treatments for melasma and complex pigmentary disorders, given their ability to fragment melanin while preserving surrounding tissue [7].

Additionally, low-fluence Nd:YAG laser protocols have demonstrated efficacy and safety in melasma management, supporting their use as part of combination strategies rather than as monotherapy [8]. In the present case, laser treatment was combined with oral tranexamic acid and topical depigmenting agents to simultaneously address multiple pathogenic pathways, including melanocyte activity, vascular involvement, and dermal pigment deposition.

The significant clinical improvement observed in this patient highlights the importance of a multidisciplinary approach in managing complex pigmentary disorders associated with systemic disease. Beyond objective pigment reduction, the marked improvement in quality of life underscores the psychosocial burden of facial hyperpigmentation and highlights the potential contribution of dermatologic management as a complementary component of systemic endocrine therapy.

This case adds to the limited literature addressing dermatologic management of Addison’s disease-related hyperpigmentation and suggests that persistent pigmentation does not necessarily indicate inadequate hormonal control. Instead, selected patients may benefit from individualized, multimodal dermatologic therapy. Further studies are needed to establish standardized treatment protocols and to evaluate the long-term safety and efficacy of laser-based and combination therapies in this patient population.

## Conclusions

This case report suggests that persistent facial hyperpigmentation secondary to Addison’s disease may benefit from a personalized, multimodal dermatologic approach. The combination of picosecond Nd:YAG laser therapy, oral tranexamic acid, topical depigmenting agents, and supportive measures was associated with clinically meaningful improvement, as evidenced by a reduction in the mMASI score from 19.8 at baseline to 7.8 posttreatment. This clinical response was paralleled by an improvement in quality of life, with the MelasQoL score decreasing from 44/70 to 20/70, without compromising systemic endocrine management. Histopathological findings suggesting overlapping features of endocrine-related pigmentation and melasma further support the need for individualized treatment strategies tailored to the underlying pathogenic mechanisms. However, given the single-case design, these findings should be interpreted with caution. Prospective studies and larger case series are warranted to better define the safety, efficacy, and long-term outcomes of combined dermatologic therapies in this patient population.
